# Etoposide and immunotherapy can improve the outcome of severe anti-GABA_B_ R encephalitis presenting with delta brush: A case report

**DOI:** 10.1097/MD.0000000000022087

**Published:** 2020-09-11

**Authors:** Wu Xiping, Xie Guomin, Wang Haifeng, Sun Qi, Zhuang Liping

**Affiliations:** Department of Neurology, Ningbo Medical Center Li-Huili hospital, Ningbo, China.

**Keywords:** anti- GABA_B_ receptor, autoimmune encephalitis, delta brush, immunotherapy, small-cell lung carcinoma

## Abstract

**Rationale:**

Anti-gamma-aminobutyric-acid B receptor (anti-GABAB R) encephalitis is clinically characterized by seizures, cognitive disorders, and behavioral changes. Most patients are diagnosed with small-cell lung carcinoma.

**Patient concerns:**

The patient suffered from a repeated grand mal seizure lasting for 10 minutes, intermittent speech vagueness, manic at night, and mental disorder.

**Diagnosis:**

The patient was diagnosed with autoimmune encephalitis. The gamma-aminobutyric-acid B(GABAB) receptor antibody test result was positive. After a bronchoscopic biopsy, the patient was diagnosed with small-cell lung carcinoma.

**Interventions:**

The patient was administered with intravenous immunoglobulin and Methylprednisolone. Etoposide was used after the small-cell lung carcinoma was diagnosed.

**Outcomes:**

After immunotherapy, following the 4 months of Etoposide and antiseizure treatment, the neurology examination revealed a remarkable improvement. MRS score reduced from 5 to 1. Electroencephalogram (EEG) recovered to normal from an extreme delta brush (EDB) electroencephalographic-pattern.

**Conclusion:**

Immunotherapy and Etoposide can improve the outcome of severe anti-γ-aminobutyric acid B receptor encephalitis with small-cell lung carcinoma. After immunotherapy and antineoplastic therapy, Electroencephalogram (EEG) can be recovered to normal from an extreme delta brush.

## 1 Introduction

Autoimmune encephalitis with an antibody against the gamma-aminobutyric-acid B receptor is usually characterized by seizures, cognitive disorders, and behavioral changes.^[1]^ Epileptic seizures are the first symptom in these patients, and most of them are temporal lobe refractory epilepsy.^[2]^ Extreme delta brush is always acknowledged in anti-NMDA receptor encephalitis but not in anti-GABAB receptor encephalitis.^[3]^ Fifty percent of anti-GABAB receptor encephalitis patients are diagnosed with small-cell lung carcinoma, which means poor prognosis.^[4]^

Here, we report a case of GABAB encephalitis with small cell lung cancer with extreme data brush. After the treatment of intravenous immunoglobulin, azathioprine, etoposide, and so on, the electroencephalogram returned to normal, the neurological function recovered, and the lung tumor significantly reduced.

## 2 Case report

The current work was conducted under the guidelines of the Ethical Committees of Ningbo Medical Center Li-Huili hospital, Ningbo, China. Written informed consent was obtained from the patient.

A 55-years-old male patient was referred to the Ningbo Medical Center Li-Huili hospital, China in 2019. More than half a month ago, the patient was unconsciousness with no induce reason, accompanied by a grand mal seizure lasting for 10 minutes. After the seizure, the patients mind returned to normal with no cold and fever, no attention was paid. Four days later, the patient had another same attack lasting for about 10 minutes and then relieved. He was sent to Ningbo Yinzhou people's Hospital for treatment. The brain MRI showed no abnormality. The chest CT showed left upper lobe space-occupying with obstructive pneumonia. Sodium valproate 500 mg twice a day was given to control the seizure. Five days ago, the patient presented intermittent speech vagueness, manic at night, and mental disorder. He was sent to Ningbo mental hospital to control the organic mental disorders. One day ago, after consultation with neurologists, the patient was sent to our hospital. Neurological examination revealed unconsciousness, uncooperative physical examination, vague speech, irrelevant answer, and no weakness on upper or lower limbs. The MRS score was 5. He had a history of hypertension for 5 years, and long-term smoking (20/day for 30 years) and alcohol use (500 ml/day for 30 years).

**Figure 1:**
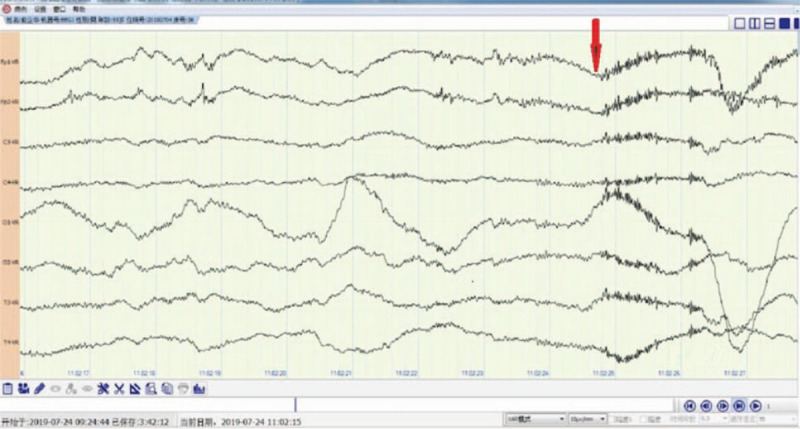
Arrow in figure shows a burst of beta frequency activity on the top of the delta wave on EEG of the patient who was coma in ICU. The EEG is 8-Lead EEG.

**Figure 2:**
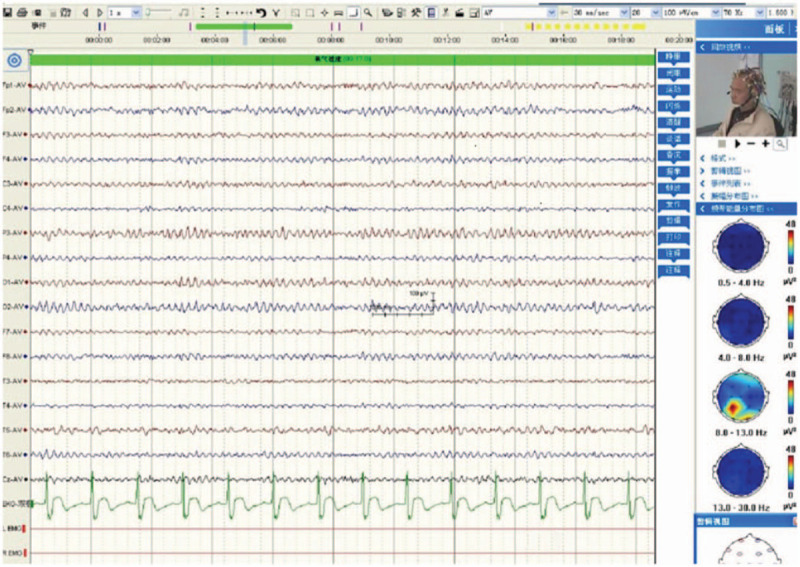
EEG in figure shows a normal wave on EEG of the patient who was recovered from GABAB encephalitis after 4 months. EEG is 16-Lead EEG.

**Figure 3:**
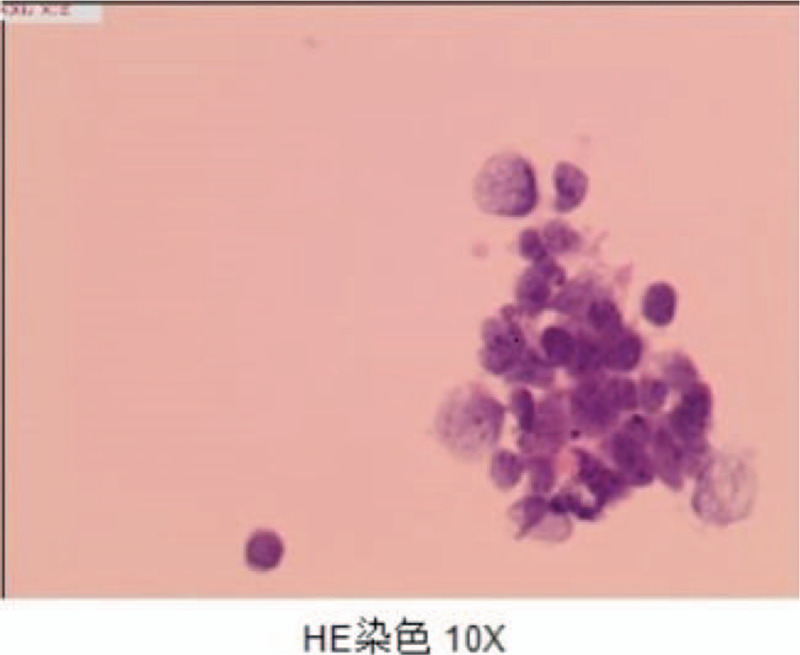
HE staining of bronchoscopic biopsy indicates small cell lung cancer.

He was sent to the intensive care unit to control the developed seizures with increasing frequency which culminated in status epilepticus. Intravenous midazolam and propofol were used. Oxcarbazepine and levetiracetam were also used. Serum and CSF were positive for GABAB receptor antibodies. Lumbar puncture showed normal opening pressures, with CSF findings as follows: nucleated cells 2 × 106/L, protein 32.7 mg/dl, glucose 3.16 mmol/L. An extreme delta brush was found using EEG (Fig. 1). Intravenous immunoglobulin and methylprednisolone were used for 5 days, followed by prednisone (60 mg/day) and azathioprine. The prednisone dose was gradually reduced every week. The patients state improved after the therapy. The seizures and mental disorders were controlled. The patient was transferred to the neurology ward. A bronchoscopic biopsy confirmed the diagnosis of SCLC (Fig. 3). Enhanced cranial MRI revealed no abnormalities. Etoposide (50 mg/day) was used to treat the SCLC. After 4 months the focus of lung cancer was reduced. There were no seizures and mental disorders. The patient could live on his own. MRS score restored to 1. EEG restored to normal (Fig. 2).

## 3 Discussion

In the past 10 years, a mounting number of autoimmune encephalitis cases have been identified. These newly defined forms of autoimmune encephalitis are concerned with antibodies against neuronal cell-surface or synaptic proteins.^[3]^ There are 6 main types of antibodies: anti-NMDA receptor (NMDAR) antibody, anti-gamma-aminobutyric acid-B receptor (GABAB) antibody, anti-leucine-rich glioma-inactivated 1 (LGI1) antibody, anti-contactin-associated protein-like 2 (CASPR2) antibody, anti-α-amino-3-hydroxy-5-methyl-4isoxazolepropionic acid 1 (AMPA1) receptor antibody, and anti-α-amino-3-hydroxy-5-methyl-4-isoxazole propionic acid 2 (AMPA2) receptor antibody.^[8]^ In all, 80% of all patients with autoimmune encephalitis are positive for antiNMDAR antibody, 7% are positive for anti-GABABR antibody, 5% are positive for anti-LGI1 antibody.^[8]^

GABAB receptors are widely distributed in the hippocampus, thalamus, and cerebellum. GABAB encephalitis presented with limbic encephalitis including seizures, cognitive impairments, confuses, and personality changes.^[1–6]^ As the most important and first symptom, epileptic seizures are always temporal lobe refractory epilepsy with a slow wave rhythm or epileptic on EEG.^[5,7]^ But the electroencephalography (EEG) of our patient shows rhythmic delta activity with a superimposed burst of beta frequency activity on the top of the delta wave (Fig. 1). This type of EEG is known as an “extreme delta brush” (EDB) pattern. It is regarded as a typical EEG for anti-NMDA encephalitis.^[3]^ Our patient is anti-GABAB receptor encephalitis and not anti-NMDA encephalitis. It means extreme delta brush can also show on the other type of acute encephalitis such as anti-GABAB receptor encephalitis. Its a response to a severe brain disease. It appears when the brain disease is severe and the patient is in an epileptic coma and disappears when the condition recovers, the epilepsy is controlled and the mind is clear. After 4 months, our patient recovered, the EEG recovered to normal (Fig. 2).

Fourty percent to 80% of patients present abnormal signals in the marginal lobe, temporal lobe, and hippocampus on MRI.^[2,5,9]^ The enhanced cranial MRI of our patient reveals no abnormalities. Other case reports^[10]^ show that F-FDG PET/CT reveals pronounced medial temporal hypermetabolism with gross hypometabolism in the rest of the brain on such brain MRI negative patient. That means brain MRI is not so sensitive to such anti-GABAB receptor encephalitis patients. With the help of PET-CT, we can better diagnose this kind of encephalitis and find the temporal lobe lesions in time.

Both serum and CSF are positive for anti- GABAB receptor antibody in our patient. The titer of anti- GABAB receptor antibody in the serum of our patient is higher than that in CSF. Other report shows that there is no difference in the positivity rate or titer of anti-GABABR antibody between CSF and serum in patients of anti-GABAB receptor encephalitis.^[8]^ That means both CSF and serum antibody should be sent to confirm a diagnose. Positive CSF or serum is helpful for diagnosis. But the titer of anti-GABABR antibody is not correlated with prognosis.^[8]^

First-line immunotherapy (intravenous immunoglobulin and methylprednisolone) is are vital in the therapy of anti-GABAB receptor encephalitis patients. Our patient has no delay in the use of first-line immunotherapy. After 4 days from his inpatient, intravenous immunoglobulin and methylprednisolone were used, and a recovered response was observed after 60 days from then on. NO seizures and no behavioral changes could be founded, only a slight cognitive deficit could be detected (MMSE score 21, Primary school culture). MRS score restored to 1. Generally, first-line immunotherapy will be initiated after a median delay of 26 days from disease onset.^[9]^ About 53% of patients will achieve seizure freedom shortly after 28 days from the start of immunotherapy.^[6]^ Although there was no delay in the treatment of immunotherapy, the patient recovered slower than most other patients. That is also a sigh of severe brain disease, which is consistent with the appearance of extreme delta brush on EEG.

Etoposide can control the tumor volume of SCLC. After 4 months, the focus of lung cancer of our patient was reduced. But 4-months follow-up time is too short, we still need a longer time to observe the recurrence and tumor deterioration. The presence of a tumor on Anti-GABAB receptor encephalitis patients is related to death.^[4]^ Most patients died from cancer progression (median survival: 1.2 years).^[9]^

## 4 Conclusion

Anti-GABAB receptor encephalitis with small cell lung cancer and extreme data brush on EEG is a severe brain disease. Early immunotherapy is crucial to control seizure and Impairment of consciousness and psychiatric disorders. After the treatment of intravenous immunoglobulin, azathioprine, etoposide, and so on, the electroencephalogram returned to normal, the neurological function recovered, and the lung tumor significantly reduced. Early immunotherapy and antineoplastic therapy are critical for improving prognosis. But there is only a case, we need more patients to confirm the conclusion.

## Acknowledgments

We would like to thank the patient for his participation in this study. We are grateful for Xiaolin Zhang from the Department of Neurology, Ningbo Medical Center Li-Huili hospital, who provided us with the guidance of English writing and publication.

## Author contributions

**Methodology:** Wang Haifeng.

**Project administration:** Zhuang Liping.

**Validation:** Sun qi.

**Writing – original draft:** Wu Xiping.

**Writing – review & editing:** Wu Xiping.
